# PrEP disclosure and discussions within social networks of people who inject drugs experiencing homelessness: a brief report

**DOI:** 10.1186/s12889-023-15153-5

**Published:** 2023-02-07

**Authors:** Leah C. Shaw, Katie B. Biello, Seamus Vahey, Jennifer K. Brody, Angela R. Bazzi

**Affiliations:** 1 Boston Health Care for the Homeless Program, Boston, MA USA; 2 Departments of Behavioral & Social Sciences and Epidemiology, Brown University School of Public Health, Providence, RI USA; 3 Center for Health Equity Research, Brown University, Providence, RI USA; 4 The Fenway Institute, Fenway Health, Boston, MA USA; 5 Harvard Medical School, Boston, MA USA; 6 Herbert Wertheim School of Public Health, University of California, 9500 Gilman Drive, MTF 265E (Mail Code 0725), CA 92093 San Diego, La Jolla, USA; 7 Department of Community Health Sciences, Boston University School of Public Health, Boston, MA USA

**Keywords:** HIV infections, Homeless persons, Substance use, Intravenous, Pre-exposure prophylaxis, Social networks, Disclosure

## Abstract

**Background:**

In the context of increasing injection-related HIV outbreaks across the United States, particularly among people who inject drugs (PWID) experiencing homelessness, there is an urgent need to expand access to pre-exposure prophylaxis (PrEP) for HIV prevention. Peer-based interventions for PrEP could be helpful for promoting PrEP uptake, yet the social experiences of using PrEP among PWID experiencing homelessness have not been thoroughly explored.

**Methods:**

To better understand social experiences surrounding PrEP use among PWID experiencing homelessness, we conducted qualitative interviews from March-December 2020 with current and former PrEP patients of an innovative, low-threshold program implemented by Boston Health Care for the Homeless Program (BHCHP) in Boston, MA. Thematic analysis of coded interview data explored participants’ perspectives and experiences with PrEP disclosure and discussions within their social networks.

**Results:**

Among interviews with 21 participants, we identified the following four interrelated aspects of their social experiences using PrEP: (1) participants’ were aware of increasing HIV transmission within their social networks, which motivated their PrEP use and disclosure; (2)  participants generally avoided disclosing their PrEP use within public spaces or casual conversations; (3)  participants expressed greater willingness to discuss PrEP with their close social contacts; and (4)  some participants self-identified as leaders or expressed interest in leading the dissemination of PrEP information within their social networks.

**Conclusions:**

Findings highlight the significance of PrEP disclosure and discussions within the social networks of PWID experiencing homelessness, suggesting a need for continued social network and intervention research—particularly to establish the feasibility and acceptability of peer-based interventions for promoting PrEP—with this marginalized population.

**Supplementary Information:**

The online version contains supplementary material available at 10.1186/s12889-023-15153-5.

## Background

People who inject drugs (PWID) represent 10% of new HIV infections in the United States annually [1]. Recent HIV outbreaks attributed to injection drug use (IDU) have occurred in multiple regions of the country, with one of the largest occurring in two communities in northeastern Massachusetts [2]. Ongoing HIV transmission in the nearby Greater Boston Area has prompted clinical advisories from local and state health departments including prioritizing screening for at-risk individuals, scaling-up prevention and treatment services, and sharing data across impacted communities [3]. Many of the hypothesized structural drivers of HIV transmission among PWID—including the pervasiveness of fentanyl in local drug supplies, limited affordable and supportive housing options, and COVID-19-related reductions in HIV testing and prevention services—continue in Boston [4], highlighting the need for expanded access to HIV prevention strategies like antiretroviral pre-exposure prophylaxis (PrEP).

Despite public health recommendations that PrEP be offered to at-risk PWID nationally and globally, uptake remains extraordinarily low, particularly among people experiencing homelessness and other structural barriers to healthcare [4]. Amidst ongoing HIV transmission among PWID, Boston Health Care for the Homeless Program (BHCHP) implemented an innovative PrEP program for PWID experiencing homelessness. An evaluation of patient medical records from this program, which involves tailored PrEP education, patient navigation, same-day PrEP prescribing, short-term prescriptions, medication storage, and intensive adherence supports including street-based daily medication dosing, suggested that it effectively increased PrEP uptake and persistence [5]. To assess the acceptability of key components of this program, we conducted qualitative interviews exploring patients’ experiences with the program and PrEP use more generally [6]. During these interviews, social experiences using PrEP (e.g., PrEP disclosure and discussions within their social networks) emerged as a key topic of importance to participants.

Social networks, which vary in size and composition, have long been recognized as central in shaping health behaviors and knowledge related to HIV transmission and prevention. In marginalized populations impacted by substance use like PWID, a large body of literature has documented the role of social networks and peer-based interventions in supporting engagement in HIV-prevention services [7]. More recently, peer-based interventions have also shown promise in preventing drug-related overdose [8]. However, despite research demonstrating trusted peers’ influence on PrEP uptake among men who have sex with men [9], little is known about PrEP disclosure or the social experiences surrounding its use among PWID. The feasibility and acceptability of peer-based PrEP interventions among PWID also remain understudied. Thus, to further inform PrEP programming and research with this understudied population, we summarize key social experiences surrounding PrEP use in a novel sample of PrEP-experienced PWID experiencing homelessness in Boston, MA.

## Methods

### Study design and sample

As detailed elsewhere [6], between March and December 2020, we conducted in-person qualitative interviews with current and former adult participants of BHCHP’s PrEP program who were purposively sampled for diversity in socio-demographic characteristics and level of engagement in PrEP services. To recruit participants, BHCHP staff described the study during routine service encounters and, if interested, introduced participants to study personnel who were present via videoconferencing on tablet computers. Interviewers screened for eligibility, which included being ≥ 18 years of age, currently experiencing homelessness (including residing in shelters or on the street), reporting past-month IDU, and being a current or former PrEP patient at BHCHP. Eligible participants provided verbal informed consent and received $25 debit cards as compensation for their time. The Boston University Medical Campus institutional review board approved study protocols and granted a waiver of documentation of consent.

### Data collection

Brief quantitative interviewer-administered surveys assessed age, race, ethnicity, sexual orientation, educational attainment, and drug use and sexual behaviors related to HIV transmission (i.e., past-month drug use, drug injection behaviors, numbers of sexual partners, frequency of condom use, and engagement in sex work; Table 1). Interviewers then conducted in-depth qualitative interviews using a semi-structured interview guide containing open-ended probes exploring HIV-related risk perceptions and behaviors, PrEP knowledge, experiences with BHCHP’s PrEP program, and general experiences using PrEP including PrEP adherence and disclosure within social networks (see Additional file 1; 6). Due to COVID-19 protocols, we conducted virtual (videoconference) or in-person interviews, which lasted ~ 30 min and were audio-recorded for professional transcription, in private outdoor locations (e.g., picnic tables in the facility parking lot). We ceased recruiting and interviewing participants after deciding (through team discussion and preliminary codebook testing, described below) that that we had reached thematic saturation regarding our primary research question, pertaining to participants’ experiences with BHCHP’s PrEP program [6].

### Data analysis

Four interviewers and a lead qualitative investigator developed a codebook collaboratively and iteratively [6]. We first independently read selected transcripts to develop potential codes and definitions. We met to discuss and compile these into a preliminary codebook that we then independently tested on additional transcripts. After comparing coding consistency, discussing discrepancies, and refining the codebook through several rounds of this process, we reached consensus on a final codebook. A trained analyst applied codes to transcripts using NVivo (v12) and discussed coding progress and emergent findings (including PrEP disclosure and discussions with peers, the focus of this paper) through regular team meetings. In-depth, thematic analysis for this paper involved a close reading and synthesis of data coded for social relationships, PrEP information and knowledge, and disclosure of PrEP use to others. We illustrate findings below using representative quotes with pseudonyms to protect confidentiality.

## Results

### Sample characteristics

Among 21 participants, median age was 35.5 years (interquartile range [IQR]: 31-37.5), 13 (62%) identified as white, four (19%) as Hispanic, and four (19%) as Black (Table 1). Fifteen (71%) identified as male, and six (29%) as female. All participants reported past-month heroin/fentanyl use and polysubstance use (primarily involving methamphetamine [*n* = 19], cocaine/crack [*n* = 18], and non-prescribed benzodiazepines [*n* = 17]). Injection frequency was high, with 12 (57%) injecting 4–9 times daily and four (19%) injecting ≥ 10 times daily. 90% of participants were staying in a shelter or on the street during the time of interview, and sixteen participants (76%) were currently taking PrEP (among whom the median duration of PrEP use was 6 weeks).

**Table 1:** Characteristics of BHCHP PrEP program participants who inject drugs and are experiencing homelessness (*n* = 21)

***Age in years, median (interquartile range; IQR)***	36 (31–38)
***Hispanic or Latino***	4 (19%)
***Racial identity***	
Black or African American	3 (14%)
White	13 (62%)
Other	5 (24%)
***Gender identity***	
Female	6 (29%)
Male	15 (71%)
***Housing, most of the time, past month***	
Street	14 (67%)
Shelter	5 (24%)
Other (e.g., motel, supportive housing)	2 (10%)
***Sexual orientation***	
Heterosexual	18 (86%)
Bisexual	2 (10%)
Homosexual or gay	1 (5%)
***Currently taking PrEP***	16 (76%)
***Median duration currently using PrEP, in weeks*** (n = 16 currently taking PrEP; IQR*)	6 (1–33)
***Median number of sexual partners, past month*** *(n = 15 sexually active participants; IQR)*	1 (2–6)
***Engaged in sex work, past month***	7 (33%)
***Frequency of condom use with sexual partners, past month*** *(n = 15 sexually active participants)*	
Sometimes/rarely/never	11 (73%)
Often/always	4 (27%)
***Drugs used, past month***	
Heroin and/or fentanyl	21 (100%)
Cocaine	19 (90%)
Crack	18 (86%)
Crystal methamphetamine	20 (95%)
Benzodiazepines (e.g., Valium, Ativan, Xanax, Klonopin)	17 (81%)
Marijuana	16 (76%)
Alcohol	8 (38%)
Gabapentin (“Johnnies”)	12 (57%)
“Street” methadone or buprenorphine (not prescribed to you)	7 (33%)
Other drugs (e.g., prescription opioids/painkillers, “ecstasy”/MDMA)	4 (19%)
***Frequency of injecting drugs, past month*** *(n = 20 with complete data)*	
10 or more times a day	4 (19%)
7 to 9 times a day	7 (33%)
4 to 6 times a day	6 (29%)
2 to 3 times a day	1 (5%)
One daily or less	3 (14%)
***Drugs injected, past month*** *(n = 20 with complete data)*	
Heroin and/or fentanyl	20 (95%)
Cocaine	15 (71%)
Crack	11 (52%)
Crystal methamphetamine	16 (76%)
***Distributive syringe sharing, past month***	
Sometimes/rarely/never	12 (57%)
Often/always	9 (43%)
***Receptive syringe sharing, past month***	
Sometimes/rarely/never	16 (76%)
Often/always	5 (24%)
***Sharing of other injection equipment (e.g., cookers, cottons, rinse water), past month***	
Sometimes/rarely/never	5 (24%)
Often/always	16 (76%)

### Social experiences surrounding PrEP use

From qualitative interviews, we identified the following four interrelated aspects of participants’ social experiences using PrEP: (1) knowledge of increasing HIV transmission within participants’ social networks motivated their PrEP use and disclosure; (2) participants generally avoided disclosing their PrEP use within public spaces or casual conversations; (3) participants expressed greater willingness to discuss PrEP with their close social contacts; and (4) some participants expressed interest in leading the dissemination of PrEP information within their social networks.

### Knowledge of increasing HIV transmission within participants’ social networks motivated their PrEP use and disclosure

Participants connected the knowledge of new HIV cases, especially those occurring among their close contacts, as an important motivating factor for their own PrEP uptake. “Jason” (pseudonym), a man in his 20’s-30’s, described PrEP as an easy way to prevent HIV in the context of his friends being newly diagnosed:

*I had friends who were comin’ up positive [for] HIV left and right [and] everybody was around here…Around Boston [HIV] was hitting hard. Everybody’s getting it…and a lot of my friends wouldn’t have [HIV] if we had just taken PrEP. You know what I mean? It’s that easy.*

“Robert,” a man in his 40’s-50’s, began PrEP after a friend was diagnosed with HIV and he heard about a cluster of new HIV cases in the local news. He described several of the new cases, who were individuals he knew well and spent time with. He actively prioritized HIV prevention after receiving this new information, including starting PrEP swiftly:

*After my buddy got infected…about six weeks later there was an article [saying] there’s now been 25 new cases of people with HIV basically the area that I hung out [in]. And I knew that I knew about 22 of those people, those cases, and so I didn’t want to become a statistic because of pure laziness or just being resistant to taking a [prevention] medication.*

These participants’ understandings of HIV transmission within their social networks and broader neighborhood motivated their PrEP uptake.

### Participants generally avoided disclosing their PrEP use within public spaces or casual conversations

Although participants expressed knowledge of HIV transmission in shared spaces, many participants described avoiding discussing or acknowledging their PrEP use in public spaces (e.g., on the street or in shelters), or during casual conversations with acquaintances or strangers. “Miguel,” a man in his 30’s-40’s, referred to his PrEP prescription as his “personal business” that was inappropriate to disclose or ask others about, explaining: “There’s no reason for me to ask somebody about PrEP; I don’t get into people’s personal [business].” He went on to explain that others became suspicious or defensive if asked about their health or PrEP status, so he generally avoided those topics while engaged in casual conversations.

Additionally, “Jose,” a man in his 30’s-40’s, explained that more routine, “regular” conversations revolved around drug use and the local drug supply, as well as other priorities such as finding food and shelter and staying physically safe from violence on the street. PrEP was not included in those everyday conversations, and “Jose” explained that having “real” conversations with other people experiencing homelessness was challenging, if not impossible:

*People don’t really tell people what [medications] they take, they don’t really talk about PrEP and Truvada here, it’s just not a conversation. People don’t get high and then say, “Oh, let’s talk about Truvada and PrEP,” you know? It’s not normally the conversations that they have here. It’s all about who has the best dope; nothing [discussed] is smart or means anything. To have real conversations around here is very hard.*

Even though participants generally avoided discussing PrEP among broader groups or acquaintances since it was considered “personal business”, some participants were more willing to acknowledge HIV prevention and risk within close social networks.

### Participants were more willing to discuss PrEP with their close social contacts

Although participants had different opinions about how and with whom they shared their PrEP experiences, most described talking with close friends and trusted peers about health-related information including PrEP services and their own PrEP use. For example, “Amber,” a woman in her 20’s-30’s, explained that the only people who knew her PrEP status were “people that matter.” Family members were included in these groups of trusted individuals for participants like “Kevin,” a man in his 20’s-30’s, who identified a cousin who was also experiencing homelessness as the reason why he started using PrEP:

*Actually, my cousin is out here, too, and she mentioned it to me. I don’t know what we were talking about, or how it came up, but I was like, “Yeah, they offered me [PrEP],” and she was like, “And you’re not taking it? You should be!” And, after that I realized it’s pretty stupid not to [take PrEP]. You know, we have something that could basically save your life.*

Multiple other participants cited their close social contacts as their primary reason for starting PrEP, and that the “people who matter” also helped them remain adherent. “Amber” described talking about HIV risk and PrEP within her tight-knight social circle, and that her partner was the reason she first started. When asked if she spoke openly about PrEP, she said:

*I don’t really like talking to too many people about it like, but like the people I’m close with, yes, like my boyfriend and friends know that I started [PrEP], you know, people that matter.*

In their conversations with close, trusted social contacts, some participants also described helping to inform their friends about local HIV transmission and PrEP in general, or explaining more specifically what PrEP was, how it worked, and potential benefits and drawbacks of using it.

### Some participants expressed interest in leading the dissemination of PrEP information within their social networks

“Robert,” who started PrEP after learning about new cases in the neighborhood, expressed knowledge about the benefits of PrEP and said, in relation to friends and close contacts, “I like to educate people about resources available to us.” When asked if he thought his discussions about PrEP with his peers could help promote their PrEP uptake, he answered:

I believe it can and has, because I’m a big proponent of PrEP…I talk to all of my buddies about it, about how easy it is to get on, and I hook them up with [the PrEP Navigator at BHCHP] and just try to educate them. There’s no reason why any of my friends that live a similar lifestyle as I do should not be on [PrEP].

Robert” also mentioned sharing information about PrEP with other participants of a nearby syringe exchange by building on his own strengths, explaining: “I am very active [there] and do whatever type of outreach I can do for PrEP because it’s my experience and [talking about] it just comes natural to me.

“Amy,” a woman in her 30’s-40’s who engaged in sex work, explained frequently educating other women and her clients about PrEP, including by sharing her own experience having an HIV-positive partner and staying HIV-negative throughout the relationship by using PrEP:

I share a lot, like when we dates and stuff, I tell [the clients] a lot about PrEP and that I think everyone should be on it. And some of them surprise me, like, “Oh, I am on it.” [And] I tell the other girls [who do sex work] about PrEP because HIV is a big thing down here…I think I’m almost selling it because I’ve never taken a pill every day like this and have it be good. But [PrEP] saved my life. And we share information with each other. It’s like a little family down here.

“Steven,” a man in his 20’s-30’s, also explained that he actively shared information about his PrEP experience within his friend group in order to motivate others to start PrEP and remain adherent. Although this type of peer-based PrEP information dissemination had not been actively promoted within BHCHP’s PrEP program, several participants seemed to enjoy this type of role and had already influenced others to start PrEP within their social networks.

## Discussion

Ongoing HIV transmission among PWID experiencing homelessness led BHCHP to develop a low-threshold program to increase PrEP access for their patient population [5]. Using data from qualitative interviews with a sample of BHCHP PrEP program participants [6], we conducted an initial exploration of the social experiences surrounding PrEP use among PWID experiencing homelessness. Although participants’ median duration of PrEP use (six weeks) was relatively short, we found that, overall, most avoided disclosing their PrEP use widely in public or large group settings, but were much more willing to share their PrEP experiences with close social contacts such as friends and family members. This could be because of trusting and intimate relationships between known persons compared to acquaintances or strangers. A minority of participants also described more actively promoting PrEP uptake among their peers, suggesting that some individuals in this population might be interested in peer- or social network-based PrEP interventions. These preliminary findings carry implications for HIV prevention intervention research with PWID experiencing homelessness. We believe such interventions could involve peer distribution of PrEP information with referrals or introductions to nearby clinical providers (i.e., acting as peer PrEP “champions”), though this warrants additional investigation. More longitudinal research with this or similar samples of PrEP patients will also be necessary, as the median duration on PrEP was relatively short in our sample.

This study adds to a very small but growing body of literature on the role of social networks in PrEP information dissemination among PWID experiencing homelessness. For example, in a study of women who inject drugs in Philadelphia, Roth et al. showed that gender homophily, similar experiences (including homelessness and perceived HIV risk), and emotional closeness were positively associated with participants’ willingness to share PrEP information with their peers [10]. Future research will be needed to investigate the feasibility, acceptability, and efficacy of peer- or social network-based PrEP interventions for PWID experiencing homelessness. Studies will also be needed to identify influential individuals (i.e., who are central and well-connected within their social networks) and the supports they will need, as studies of “peer change agents” or “PrEP champions” have identified challenges with re-traumatization, burnout, and need for refresher trainings or additional supports [8, 9]. Beyond disseminating PrEP information and connecting peers with PrEP services, studies engaging PWID experiencing homelessness should investigate how “PrEP change agents” could help support adherence (for daily oral PrEP) and retention in care (for longer-acting PrEP modalities).

There are limitations to this exploratory study. First, our findings are based on a small sample of participants recruited from a single organization in a unique geographical and socio-political context: Massachusetts benefits from near universal healthcare, and Boston enjoys relatively strong financial support for public health initiatives and research, limiting generalizability to other populations. Second, the self-report nature of our interviews may have introduced recall and social-desirable bias. Additionally, the overall study that generated data presented here focused on evaluating BHCHP’s PrEP program [6]; although participants’ social experiences surrounding PrEP use emerged as an important topic, we may have missed opportunities to more systematically inquire about specific aspects of these experiences (e.g., PrEP or addiction-related stigma) that could be relevant for intervention development. We call for future research to better explore the relationships between HIV knowledge and PrEP disclosure and potential social network-based interventions to support PrEP information dissemination and uptake among PWID experiencing homelessness.

## Conclusions

Despite these limitations, this brief report is among the first study, to our knowledge, to qualitatively explore PrEP disclosure and discussions within the social networks of PrEP-experienced PWID experiencing homelessness. Although most participants were hesitant to disclose their PrEP use openly in public settings, many discussed PrEP with more trusted, close social contacts, and importantly, a minority enjoyed playing more active roles in educating and motivating their peers to take up PrEP. Peer- or social network-based HIV prevention interventions warrant additional investigation in this at-risk population with historically low access to and uptake of PrEP.

## Supplementary Information

**Additional file 1.** Qualitative Interview Guide for Participants. 

## Data Availability

Coded, de-identified data will be made available upon reasonable request to the corresponding author.

## Abbreviations

PWID: People who inject drugs
PrEP: Pre-exposure prophylaxis
BHCHP: Boston Health Care for the Homeless Program
IDU: Injection drug use
IQR: Interquartile range
