# Supplementary material for: PrEP disclosure and discussions within social networks of people who inject drugs experiencing homelessness: a brief report
Source: BMC Public Health. 2023 Feb 7;23:263. doi: 10.1186/s12889-023-15153-5 (PMC9903274; doi:10.1186/s12889-023-15153-5)
Supplement: Supplementary file 1 — Additional file 1. Qualitative Interview Guide for Participants. [file 12889_2023_15153_MOESM1_ESM.pdf]

BHCHP PrEP Study

Qualitative Interview Guide for Participants

PREAMBLE: [READ ALOUD]: Hi, my name is **[interviewer]** and I'm an interviewer for this project. Do you have any questions before we begin? **[TURN ON RECORDER; READ]:** This is **[interviewer]** conducting a **[participant]** interview with **[PID]** on **[date]** at **[time]** at **[location].**

1. **To get started, can you tell me a bit about what your drug use is like in a typical week?**

   a. What kinds of drugs do you generally use? What kinds of drugs do others people you know use?

   b. Do you feel that you are at risk for getting sexually transmitted infections or HIV? Why (or why not)?

2. **What are the health problems or concerns that are the most important to you right now? What do you worry about the most?**

3. **Tell me about your experience accessing health or other services for people who use drugs through BHCHP?**

   a. What kinds of services you have used recently?

   b. How helpful were these services?

   c. How have your interactions been with the people providing these services? Does this influence your decisions to seek care?

4. Today I would like to ask you about the HIV prevention pill called pre-exposure prophylaxis, or PrEP for short. Some people also call it Truvada or the "HIV prevention pill." **Can you tell me what you know about PrEP?**

5. I'd like to hear more about your experiences using PrEP. **Tell me about how you first learned about PrEP.**

6. **How did you decide to start using PrEP?**

7. We are interested in improving the PrEP program at BHCHP that helps people like you use PrEP, and I'd like to hear about your experiences at different points in the process. **Can you tell me what it was like to get started using PrEP?** Walk me through that process.

   a. Was there anything about your experience getting on PrEP that you wish had gone differently?

8. **IF CURRENTLY OR FORMERLY TAKING PrEP:** Importantly, to be effective in HIV prevention, PrEP must be taken every day and continued as long as someone needs protection. **Was it easy or difficult for you to do this? Why?**

    a. What kinds of things made it challenging for you to take PrEP daily?

    b. What did you do about these challenges? What are some things you did (or still do) to remember to take PrEP daily?

    c. Has the BHCHP PrEP program helped you remember to take the medication daily? If so, how?

9. **IF STOPPED TAKING PrEP AT SOME POINT: Why did you stop taking PrEP?** Did you ever stop and restart? Why?

    a. Has BHCHP helped you stay involved in healthcare?

10. We've heard that a lot of people don't know about PrEP, what it is, or how to get it. **How do you think we could reach more people like you with information about PrEP and how to get on it PrEP?**

    Probes: informational materials (content, locations, etc), info sessions at local CBOs (provide local examples, combine with overdose trainings), trusted sources of health information (outreach workers, peers/word of mouth)

11. As someone who has considered PrEP (or are on it now/in the past), **what was the single most important thing that made you think seriously about taking it?** Is there anything else that might have worked even better?

12. Some programs use cell phones or the internet to keep in touch with patients and help remind them about appointments and taking their medications. **Would it have been helpful if someone from here could have contacted you via phone/internet to remind you about your upcoming apiontments or taking your meds, or just to check in?**

    a. Do you have a cell phone or smart phone?

    b. Do you have regular access to the internet?

CONCLUSION

OK, please let me check to make sure I covered everything. **[NOTE: check over interview guide for any missed questions to ask.]** Thank you for sharing your experiences and opinions with me. Is there anything else that you'd like to tell me about these topics?

Great, this concludes the interview. Thank you again for taking the time to participate and share all of this valuable information with us. Please get in touch with me if you have questions later.

[TURN OFF TAPE RECORDER]
